# The effects of simulated +Gz and microgravity on intervertebral disc degeneration in rabbits

**DOI:** 10.1038/s41598-019-53246-7

**Published:** 2019-11-12

**Authors:** Di Wu, Xi Zhou, Chao Zheng, Yu He, Lingjia Yu, Guixing Qiu, Zhihong Wu, Ji Wu, Yong Liu

**Affiliations:** 1 Department of Orthopedic Surgery, Peking Union Medical College Hospital, Peking Union Medical College and Chinese Academy of Medical Sciences, Beijing, China; 2 Department of Orthopedic, Air Force General Hospital of the Chinese People’s Liberation Army, Beijing, China; 3 Department of Plastic Surgery, Plastic Surgery Hospital, Chinese Academy of Medical Sciences & Peking Union Medical College, Beijing, China; 4 Department of Orthopedics, Beijing Friendship Hospital, Capital Medical University, Beijing, China; 5 Beijing Key Laboratory for Genetic Research of Skeletal Deformity, Beijing, China

**Keywords:** Physiology, Astronomical instrumentation, Diseases

## Abstract

The overall objective of this study was to test the hypothesis that +Gz (hypergravity/positive acceleration) and microgravity can both aggravate intervertebral disc degeneration (IVDD). Due to **+**Gz and microgravity, many pilots develop IVDD. However, the lack of animal models of IVDD under conditions of simulated **+**Gz and microgravity has hampered research on the onset and prevention of IVDD. Rabbits were randomly allotted to a control group, microgravity group, **+**Gz group, or mixed (**+**Gz + microgravity) group. A tail-suspension model was utilized to simulate a microgravity environment and an animal centrifuge to mimic **+**Gz conditions. After exposure to the above conditions for 4, 8, and 24 weeks, the body weights (BW) of animals in the control group gradually increased over time, while those of animals in the microgravity and mixed groups both decreased (*p* < 0.001). As compared with the control group, the proteoglycan content of animals in the other three groups was significantly reduced (F = 192.83, *p* < 0.001). The imageological, histopathological, and immunohistochemical changes to the L6–S1 intervertebral disc samples suggests that the effects of **+**Gz and microgravity can aggravate IVDD over time. The mixed effects of **+**Gz and microgravity had the greatest effect on degeneration and **+**Gz had a particularly greater effect than microgravity.

## Introduction

Everyone can experience gravitational forces when riding in an elevator, roller coaster, etc. The influence of microgravity and hypergravity on living systems, such as neuronal, thyroid, and tendon cells, has attracted significant attention^1–3^. Gravity influences physical and biological processes and plays a critical role during the development and homeostasis of human tissues. The rate of intervertebral disc degeneration (IVDD) is approximately quadrupled among pilots and astronauts following spaceflight because of exposure to the specific environmental conditions of microgravity and hypergravity^4,5^. A survey by Rabin *et al*.^6^ of 722 pilots and astronauts employed by the National Aeronautics and Space Administration found that 384 (53%) had low back pain. Analysis conducted by the Japanese Aeronautical Laboratory of 260 pilots found that 12.3% had IVDD^7^. Dagenais *et al*.^8^ analyzed 147 studies conducted in different countries from 1997 to 2007 and found that low back pain caused by IVDD was a major drain on financial and medical resources.

IVDD onset is characterized by a reduction in the number of disc cells and decreased ability to bind water due to proteoglycan (PG) decomposition in the nucleus pulposus (NP). Meanwhile, the layered structure of the annulus fibrosus (AF) begins to deteriorate, resulting in the development of internal fissures that spread around the periphery of the AF^9^. Collagen-1, which is mainly present in the AF, while collagen-2 is mainly present in the NP, forms a small loose fibrous network associated with PG that maintains the stability and strength of the NP matrix^10^. Under normal physiological conditions, transforming growth factor beta (TGF-β) is a protective factor that inhibits the degradation of collagen and other components of the extracellular matrix, promotes the repair of intervertebral disc (IVD) tissue, and even reverses degeneration of the NP and AF. In IVDD, the content of collagen-1 and TGF-β is increased, while that of collagen-2 is decreased, accompanied with PG degradation^11^.

Although the exact pathogenesis of IVDD remains unclear, the roles of aging, genetic susceptibility, nutritional disorders, mechanical load, and other factors have been widely acknowledged^12^. Many studies have investigated the impact of microgravity on skeletal muscles; however, most have focused on bone loss rather than IVDD^13–15^. To the best of our knowledge, no study has focused on the cumulative and interaction effects of both +Gz (positive acceleration) and microgravity, which are thought to be closely related to the degree and speed of IVDD^16^. Therefore, the present experiment simulated conditions of +Gz and microgravity in order to test the hypothesis that +Gz and microgravity can both aggravate IVDD.

**Figure 1:**
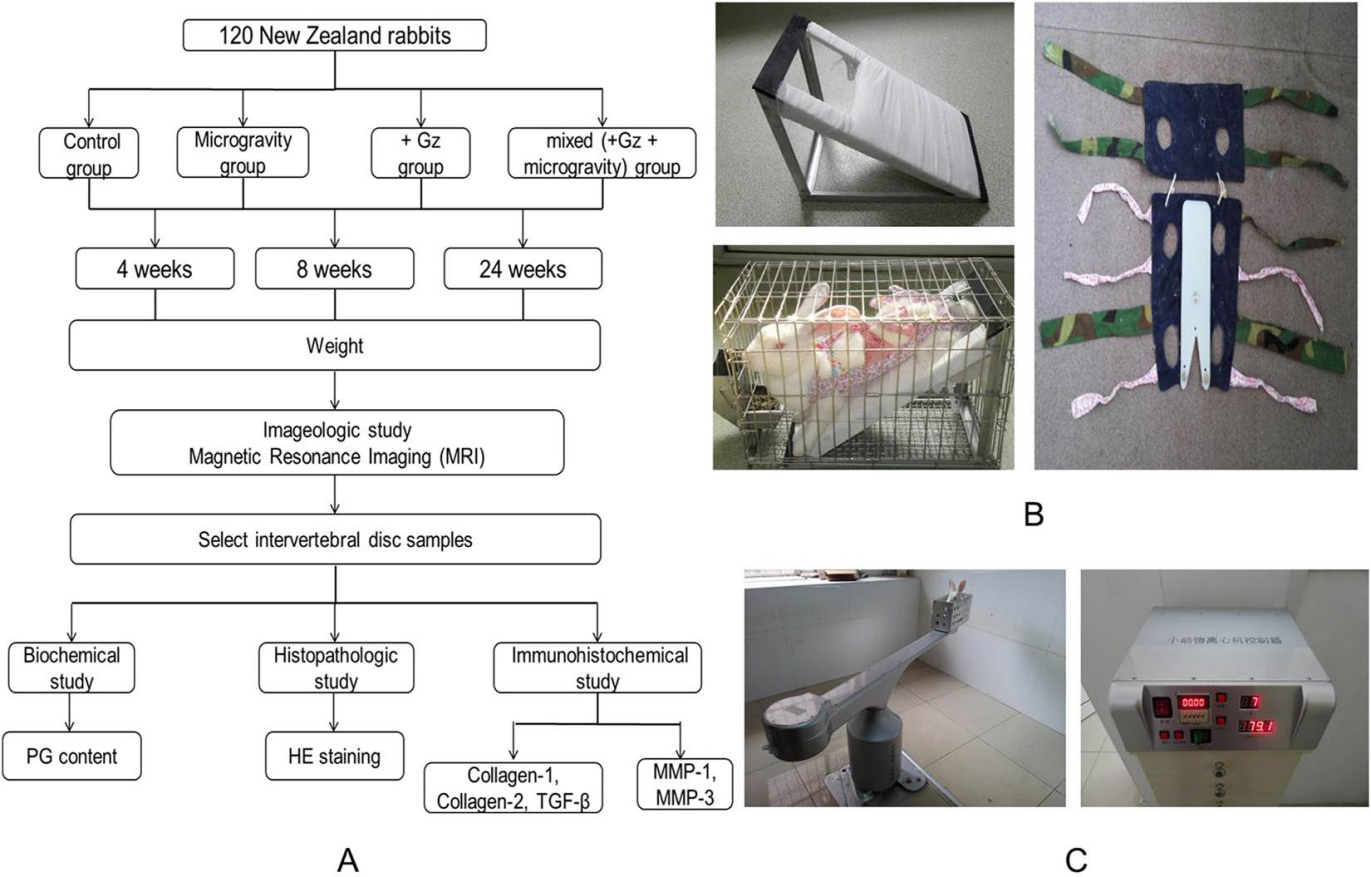
(**A**) Timeline of the experimental procedures. Discs were collected from 10 rabbits in each group at 4, 8, and 24 weeks. (**B**) The tail-suspension model was used to simulate microgravity condition. (**C**) A high-speed animal centrifuge was used to simulate +Gz condition. The speed of the centrifuge was set to 79.1 rpm to obtain +7 Gz.

To examine the impact of microgravity or hypergravity on a living body, many animal experiments have been performed aboard the International Space Station^17^. However, due to high costs and limited resources, performing experiments in actual +Gz and microgravity environments is not feasible. An improved tail-suspension model was utilized to simulate a microgravity environment^18^ and an animal centrifuge to mimic +Gz conditions^19^. Using up-to-date technologies, including recording of changes in body weight (BW), imageological, biochemical, histopathological, and immunohistochemical studies were conducted to systematically explore the effects of simulated +Gz and microgravity on IVDD (Fig. 1).

## Results

### General conditions of animals

Throughout the experiment, only a small number of the experimental animals exhibited signs of anorexia, diarrhea, listlessness, or infection. To maintain the overall health of the experimental animals, a series of measures were employed, which included actively improving the feeding environment and regular use of prophylactic antibiotics. There were significant differences in the BWs of the rabbits among the four groups at all time periods. The BWs of animals in the control group gradually increased over time (F = 484.76, *p* < 0.001), while those of animals in the microgravity group (F = 66.02, *p* < 0.001) and mixed (+Gz + microgravity) group (F = 212.09, *p* < 0.001) both decreased. Meanwhile, +Gz had no effect on the BWs of the rabbits in the +Gz group (F = 0.07, *p* = 0.98). As shown in Table 1, with an increase in exposure time (from week 4 to 24), the effect of microgravity significantly decreased the BWs of the rabbits and the mixed effects of +Gz and microgravity had the greater effect on BW loss.

**Table 1:** Body weight changes in each group.

ody weight (kg)	0 week	4 weeks	8 weeks	24 weeks	F-value	P-value
Control group	3.13 ± 0.09	3.62 ± 0.13	4.22 ± 0.18	6.34 ± 0.33	484.76	<0.001
Microgravity group	3.18 ± 0.09	3.05 ± 0.10	2.92 ± 0.09	2.64 ± 0.10	66.02	<0.001
+Gz group	3.17 ± 0.08	3.18 ± 0.08	3.17 ± 0.07	3.16 ± 0.51	0.07	0.98
Mixed group	3.16 ± 0.06	3.04 ± 0.06	2.91 ± 0.06	2.41 ± 0.10	212.09	<0.001
F-value	0.79	83.48	305.58	1057.56		
P-value	0.51	<0.001	<0.001	<0.001		

### Imageological changes of IVD

**Figure 2:**
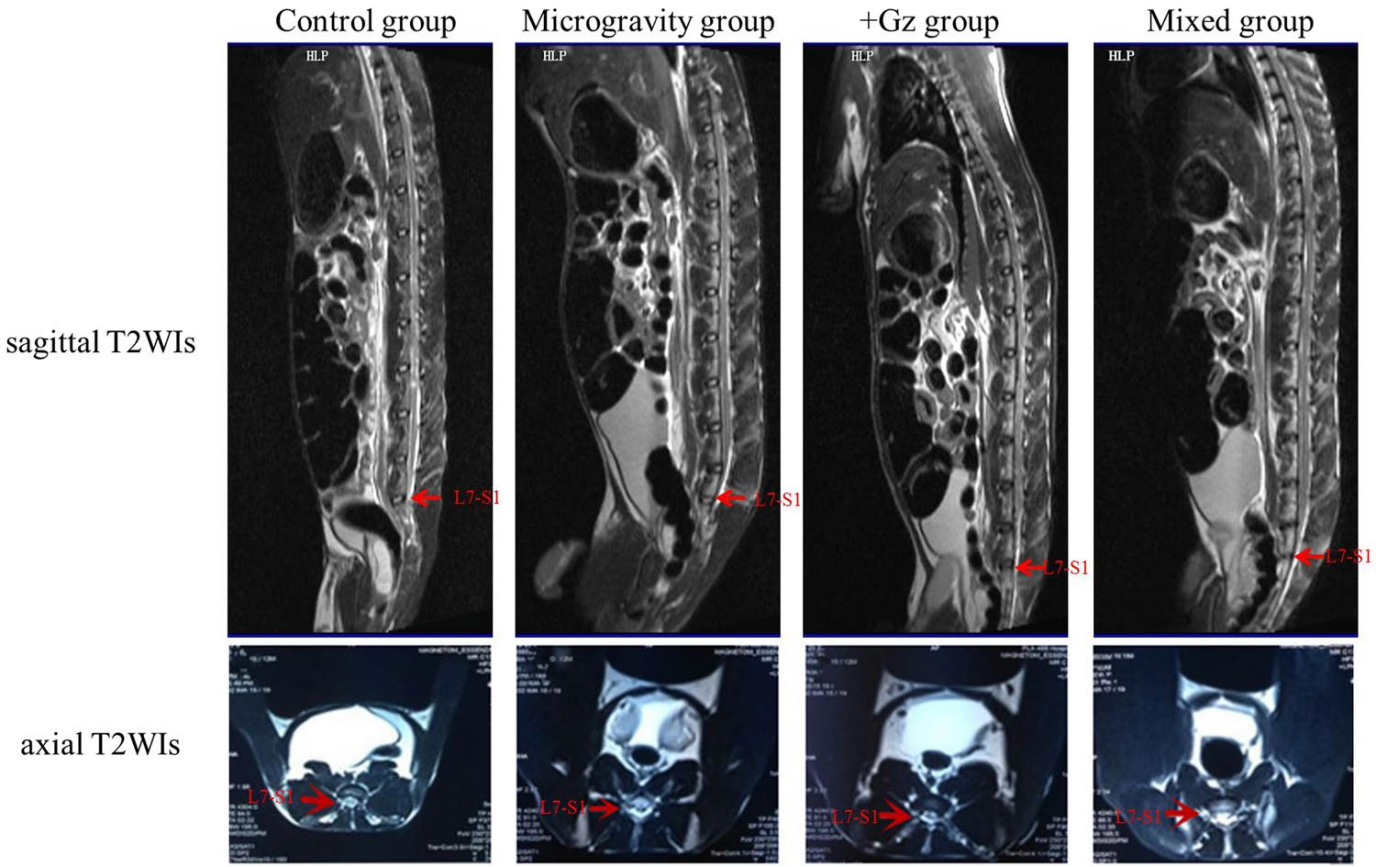
Imageological results of animals in each group. As shown by the sagittal T2WIs at 24 weeks, the IVD at L7–S1 of one animal in the control group was classified as Pfirrmann grade I (red arrow). The structure of the disc is homogeneous with a bright hyperintense white signal intensity and a normal disc height. Similarly, the IVD at L7–S1 of one animal in the microgravity group was classified as grade II (red arrow). The structure of the disc is inhomogeneous with a hyperintense white signal. The distinction between the NP and AF is clear, and the disc height is normal, with or without horizontal gray bands. Grade III (red arrow) in the +Gz group: The structure of the disc is inhomogeneous with an intermediate gray signal intensity. The distinction between the NP and AF is unclear, and the disc height is normal or slightly decreased. Grade IV (red arrow) in the mixed group: The structure of the disc is inhomogeneous, with a hypointense dark gray signal intensity. The distinction between the NP and AF is lost, and the disc height is normal or moderately decreased. As shown by the axial T2WIs, spinal stenosis developed in all of the experimental groups, but not the control group.

MRI showed different degrees of signal intensities depending on the different tissues embedded in the vertebral body marrow and spinal cord, while signals of the intervertebral disks on sagittal and axial T2WIs indicated the level of lumbar IVDD according to the grading measurements of the Pfirrmann category. At weeks 4, 8, and 24, there was no indication of narrowing of the disc space, modic changes (a common observation by MRI marked by signal intensity changes in vertebral body marrow adjacent to the endplates of degenerating discs), disc herniation, or spinal stenosis among the animals in the control and microgravity groups. MRI revealed narrowing of the intervertebral spaces in seven rabbits in the +Gz group and nine in the mixed group. At week 24, modic changes and water content decreases in the discs were observed in 40% and 80% of rabbits in the +Gz group, and 40% and 100% of rabbits in the mixed group, respectively (Fig. 2). The results showed that the effect of +Gz plays an important role in IVDD over time.

### Changes in PG content in IVDs at L6–L7

**Figure 3:**
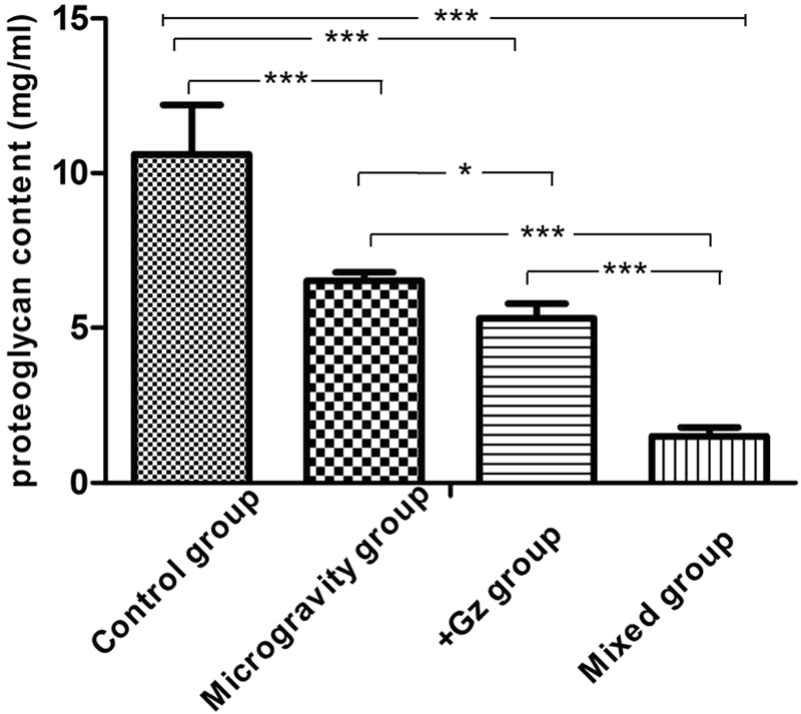
Quantitative analysis of PG content (mg/mL) in L6–L7 IVD of each group at 24 weeks. Data are presented as the mean ± SD. *p < 0.05, ***p < 0.001.

To evaluate the effects of +Gz and microgravity on PG content, changes in PG content in IVDs at L6–L7 were compared among all groups. At week 24, the PG content dramatically decreased, as compared with that in the control group. Results of phloroglucinol method^20^ interactively indicated that the PG content of animals in other groups was significantly reduced (F = 192.83, *p* < 0.001) (Fig. 3).

### Pathological changes to IVDs at L7–S1

**Figure 4:**
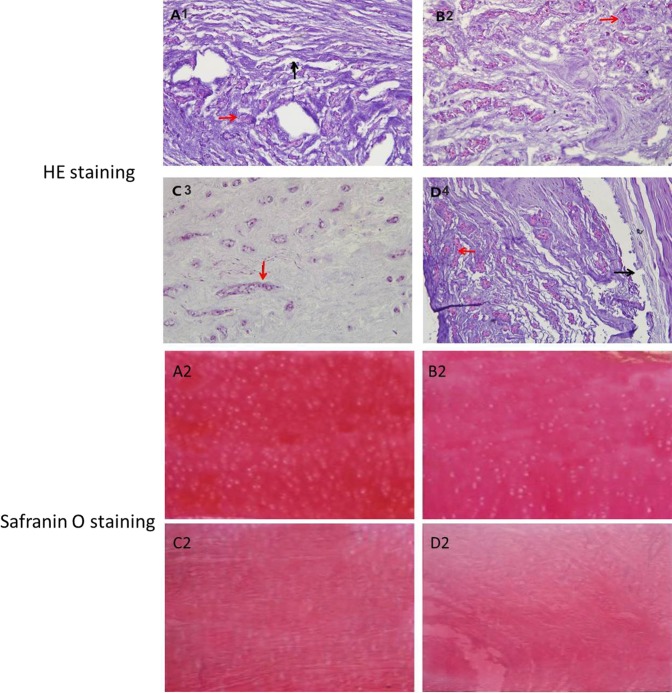
HE staining (×400) of L7–S1 IVD of each group at 24 weeks. Part of the NP was absent (red arrow) and the inner layer of the AF was slightly distorted (black arrow) in the control group (**A1**). The NP cell content decreased and rounded chondrocytes occasionally appeared (red arrow) in the microgravity group (**B1**). The NP cell content decreased significantly with metaplasia of chondrocytes (red arrow) in the +Gz group (**C1**). NP cells were scattered (red arrow) with internal fissures in the AF (black arrow) in the mixed group (**D1**). Safranin O staining (×400) was significantly stronger in the control group (**A2**), as compared to the three experimental groups, and there were no significant differences between the microgravity group (**B2**) and the +Gz group (**C2**). The mixed group showed the lightest color (**D2**).

To determine the effects of +Gz and microgravity, the pathological changes of IVDs at L7–S1 at weeks 4, 8, and 24 were analyzed by HE staining. At weeks 4 and 8, observation by light microscopy indicated that most NPs were complete and had a clear boundary with the AF, while NP cells were evenly distributed and loose reticular fibers formed throughout the extracellular matrix. At week 24, a few tissue sections in the control group showed partial absence of NPs and formation of cell clusters, while the inner layer of the AF was slightly distorted. Furthermore, internal fissures were observed with the occasional appearance of rounded chondrocytes in the NPs of animals in the microgravity group. There were fewer and scattered NP cells in sections obtained from animals in the +Gz and mixed groups, accompanied with metaplasia of chondrocytes. The number of NP cells was significantly reduced and fissures existed between the inner and outer layers (Table 2). NP cells and PG from all the groups were stained with safranin O, which exhibited a red color. Safranin O staining was significantly stronger in the samples from the control group, as compared with the other three groups. There was no significant difference in safranin O staining between the microgravity and +Gz groups. In the mixed group, safranin O staining was the lightest (Fig. 4).

**Table 2:** Nishimura grading results of lumbar intervertebral disc degeneration in each group at 24 weeks.

Numbers of rabbits	Nishimura I	Nishimura II	Nishimura III	Nishimura IV	Nishimura V
Control group	8	2	0	0	0
Microgravity group	1	8	1	0	0
+Gz group	2	1	7	0	0
Mixed group	0	0	1	8	1

Pathological changes of intervertebral discs were classified by Nishimura gradings. Nishimura 0, normal; Nishimura I, mildly serpentine with rupture; Nishimura II, moderately serpentine with rupture; Nishimura III, severely serpentine with mildly reversed; Nishimura IV, severely reversed contour; or Nishimura V, indistinct.

### Changes in collagen-1, collagen-2, TGF-β and matrix metallopeptidase (MMP) concentrations in IVDs at L7–S1

**Figure 5:**
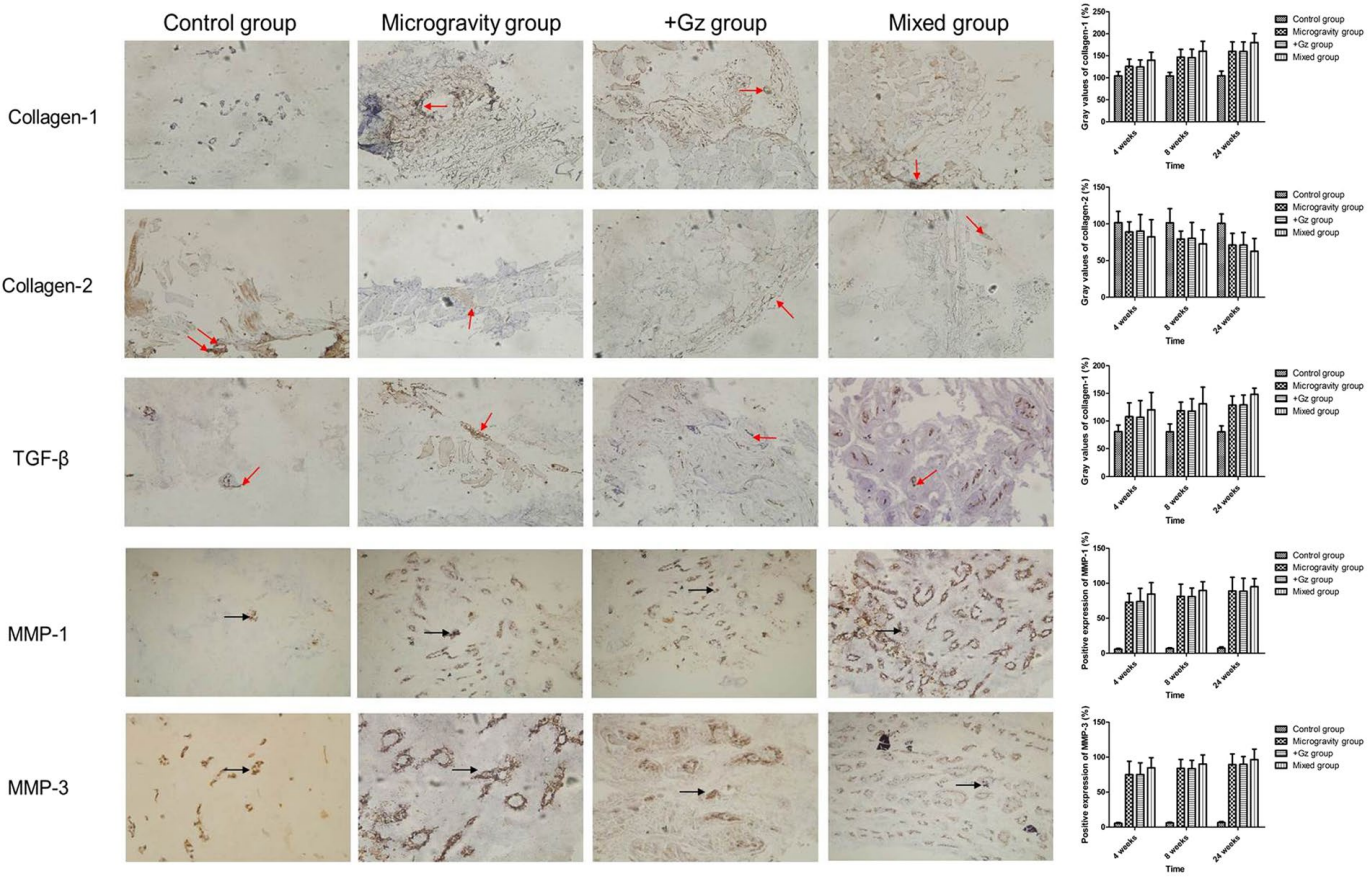
Immunohistochemical results observed by optical microscopy (×400) of the IVDs at L7–S1 of different groups at 24 weeks. Positive granularity of collagen-1, collagen-2, and TGF-β were all clay brown or brownish yellow (red arrow). Gray values (%) of collagen-1, collagen-2, and TGF-β in the IVDs at L7–S1 of different groups. Positive cells are indicated by brown-yellow or brown particles in the cytoplasm and blue-stained nuclei (black arrow). Data are presented as the mean ± SD.

Next, the effects of +Gz and microgravity on the expression levels of collagen-1, collagen-2, and TGF-β in IVDs at L7-S1 were examined. Positive granularity of collagen-1, collagen-2, and TGF-β were all clay brown or brownish yellow and located in the AF and NP. Immunohistochemical results at 4, 8, and 24 weeks showed significant upregulation of both collagen-1 and TGF-β in the microgravity group, +Gz group, and mixed group (*p* < 0.001), but not the control group (collagen-1: *p* = 0.991 and TGF-β: *p* = 0.996). As compared with the control group, collagen-1 and TGF-β levels were gradually upregulated in both AF and NP over time, with significantly increases at 8 and 24 weeks. On the contrary, collagen-2 was gradually downregulated (*p* < 0.001) over time in the three experimental groups, but not the control group (*p* = 0.991). There were few positive cells in the control group at all time periods and there was no significant difference between the time points (MMP-1: *p* = 0.126 and MMP-3: *p* = 0.134). Positive expression rates in the microgravity, +Gz, and mixed groups increased gradually with prolonged exposure time, with significant differences between time points in each group (*p* < 0.001). The strongest expression was in the mixed group at all time points, followed by the +Gz group, and the microgravity group (Fig. 5).

## Discussion

Using a centrifuge and tail-suspension model designed for rabbit studies, the effects of +Gz and microgravity on IVDD were systematically explored. Given that acquiring and maintaining rats and rabbits is easier and more affordable than that of other animals, recent studies of IVDD have mostly used the rat^17,19^. However, the rabbit is much larger than the rat, with morphologic characteristics that are more similar to those of humans^21,22^.

Analysis of the changes in BW of the experimental animals found that weight loss was mainly caused by severe restriction of physical activities induced by tail-suspension, while the animals’ resistance led to more physical compression, and the posture of the head downward and feet upward had harmful effects on digestion and absorption of food, as well as breathing and circulation^23^.

The normal NP and the medial side of the AF in the IVDs had high signal intensities on sagittal T2WIs, and with the development of IVDD, the water content of the NP was reduced, which resulted in weakening of the signal intensity^24,25^. In combination with the Pfirrmann’s grading system showed no significant short-term degeneration in the lumbar IVDs. In the long-term (24 weeks), IVDD was occasionally observed. Similarly, several rabbits in the microgravity group were graded as level II at 24 weeks due to the effect of microgravity. Although +Gz alone and in combination with microgravity can accelerate degeneration in the early stage (4 weeks), with an increase in exposure time, disc degeneration becomes more obvious. It is worth mentioning that the assessment of disc degeneration in the early stage was not observed by MRI, but was only confirmed by histological examination^26^. As IVDD is initiated by histomorphological and molecular changes, only when the degeneration achieved a certain degree, imageological changes can be depicted. Besides, pathological performance at the micro level is easier to detect early, while imageological examinations are macroscopic.

The healthy NP is rich in PG and collagen-2, whereas the AF is rich in collagen-1^9,26,27^. Collagen-1 and collagen-2 are the most abundant collagens in discs, accounting for about 70%–80% of all collagen. Previous studies suggest that members of the TGF-β family have pleiotropic effects on IVD cells^28^. Yang *et al*.^29^ found that the TGF-β content was significantly increased in degenerative disc disease. Jin *et al*.^30^ revealed that TGF-β was a protective factor of NP and AF, regulated collagen-1 and collagen-2, and inhibited or even reversed the process of IVDD^31,32^. The present experiment showed the contents of collagen-1, collagen-2, and TGF-β did not change significantly with no intervention, and there was no significant degeneration of the NP and AF in the control group, while under the conditions of +Gz and/or microgravity, the contents of collagen-1 and TGF-β gradually increased and that of collagen-2 decreased with an increase in exposure time. Meanwhile, the degeneration of NP and AF were positively correlated with exposure time.

Humans retained notochordal cells in ageing discs, the number is very low. In comparison, rabbit discs have a high number of notochordal cells^33^. It was shown that notochord cells can effectively maintain the size of the NP and prevent disc degeneration, which was often caused by the disappearance of notochord cells in animal models^34^. Our experiment showed that the cell phenotype had changed in the central region of NP, which probably resulted from the phenotype changes of notochordal cells, or caused by the growth of fibrocartilaginous tissue from cartilage endplate. These changes reduced the hydration ability of intervertebral disc and the resistance to degeneration, which can be considered as the initial changes of IVDD.

What determines the mechanisms by which +Gz and/or microgravity induces disc degeneration? The substance and energy metabolism of IVDs is maintained by osmosis because of the lack of a direct nutrient supply^5^. Many scholars have acknowledged that this nutrient delivery method is required by the vertebrae to bear loads and to maintain normal hydrostatic pressure between the vertebrae, thereby further ensuring the distribution of body fluids and blood, as well as normal metabolism among the discs and vertebrae^35–38^. Under conditions of +Gz or/and microgravity, IVDs and vertebrae had to bear loads that were much greater than physical hydrostatic pressure, resulting in weakened permeability of cartilage and endplates, and subsequent dramatic reductions in the nutrient supply and metabolism of NP and AF, especially oxygen and glucose supplies, thereby reducing the synthesis and concentration of PG in the NP, causing dehydration and atrophy of the IVD tissue, and hindering the repair and reconstruction of NP and AF, ultimately accelerating the progression of IVDD. Besides, it has been confirmed that with the changes of pressure and stress on the IVD, interleukin 1 (IL-1), tumor necrosis factor, MMPs, and other serum inflammatory factors can mediate and accelerate the process of IVDD, and their expression is regulated by a series of signal channels, such as the mitogen-activated protein kinase pathway, nuclear factor-κB pathway, and Wnt/β-catenin pathway^39^.

In conclusion, the conditions of simulated +Gz and microgravity can be widely applied to studies of the causes and prevention of IVDD. The results of this study suggest that the mixed effects of +Gz and microgravity were more obvious than the single impact of +Gz or microgravity alone, which could accelerate IVDD. Besides, the degree of degeneration was positively correlated with exposure time. Further neurologic function testing should include other spinal segments and consider secondary injuries.

## Methods

### Ethics statement

The study protocol was approved by the Ethics Committee of Peking Union Medical College Hospital, Peking Union Medical College and Chinese Academy of Medical Science, and all animal experiments were performed in accordance with the guidelines of the Animal Care and Use Committee of Peking Union Medical College Hospital.

### Rabbits

A total of 120 healthy New Zealand male white rabbits, 7–8 months old (median: 7.6 months), weighing 3.0–3.5 kg (median: 3.1 kg), were purchased from the Experimental Animal Center of the First Affiliated Hospital of Chinese People’s Liberation Army (Beijing, China). The rabbits were assigned to one of four groups of 30 rabbits each: a control group, microgravity group, +Gz group, or mixed group. According to the principle of statistical reliability, if an experimental animal was dead or missing, a new experimental rabbit was added.

### Description of the devices for simulating microgravity and +Gz

An improved tail-suspension model, based on the mouse model proposed by Wronski and Morey-Holton^18^, was utilized to simulate microgravity, where the hind limbs were hung with no weight bearing on the fore legs to maintain the body in a horizontal plane at 45° with a soft cushion (suspensory weskit) to protect the abdomen (Fig. 1B). The +Gz environment was induced by centrifugation of a custom-made mesh rotating box with a 1.6-m arm (YL-001; Apos Optoelectronic Corp., Changchun, China) at 79.1 rpm to obtain + 7 Gz. Each animal was fixed in the box and forced to the axis by high-speed centrifugation (Fig. 1C).

According to the daily training load to pilots and astronauts^5,22,40^, the physical capacity of rabbits, and the outcomes of preliminary experiments, a hypergravity load of +7 Gz was selected. The results of the present study showed that after exposure to +7 Gz three times for 60 s each with 10-min intervals, the performance of the rabbits was generally normal. The tail-suspension method to simulate weightlessness or microgravity has been generally recognized as both non-invasive and reliable^17,18^. Due to the shorter tail and average size of New Zealand white rabbits, the rabbits were suspended by the tail and hind legs. This improvement to the tail-suspension method achieves a simulated microgravity condition, but also improved the experimental environment, which was conducive to survival and long-term observation of the experimental animals.

### Procedures

To maintain the overall health of the experimental animals, a series of measures were employed, which included actively improving the feeding environment and regular use of prophylactic antibiotics. All rabbits were housed in a light- and temperature-controlled environment with free access to food and water, and were weighed weekly. The control group received no intervention. Rabbits in the microgravity group were suspended for 5 days per week, while the others for two days. Those in the +Gz group were exposed to +7 Gz three times for 60 s each at 10-min intervals and rest at other times, which was repeated every other day. The rabbits in the mixed group were also suspended for 5 days as with the microgravity group and exposed to +Gz following in the same manner as in the +Gz group. Ten rabbits were randomly chosen and assigned to subgroups at 4, 8, and 24 weeks after weighing. Simulation of +Gz and microgravity were performed at the Air Force Institute of Aviation Medicine (Beijing, China). Imageological, biochemical, histopathological, and immunohistochemical studies were conducted at the Key Laboratory of Peking Union Medical College Hospital.

### Imageological study

At 4, 8, and 24 weeks, all animals were anesthetized with ketamine at a concentration of 10 mg/kg BW and underwent magnetic resonance imaging (MRI) examinations. MRI was performed using a 3.0 T clinical magnet (Siemens AG, Munich, Germany) to observe structural changes on sagittal T2-weighted images (T2WIs) (repetition time = 3200.0 ms; echo time = 112.0 ms). The T2WIs were evaluated according to the classification of IVDD as reported by Pfirrmann *et al*.^41^. All images were evaluated by another orthopedist blinded to the study conditions.

### Selection of IVD samples for biochemical, histopathological, and immunohistochemical studies

The rabbits were immediately sacrificed upon completion of the imageological examination. IVD samples of L6–L7 and L7–S1 were obtained with a sharp scalpel, then fixed in 10% paraformaldehyde and stored at room temperature. The reason for collecting IVD samples from L6–L7 and L7–S1 was to obtain samples subjected to the largest load, as changes at these locations were the most obvious. According to a report by Phillips *et al*.^42^, the caudal vertebrae (L7–S1) usually sustained a greater load than the proximal vertebral segments (L7–S1 > L6–7 > L5–6 > L4–5). Therefore, IVD samples from L6–L7 and L7–S1 were selected for key observations and evaluations of the range of interest.

### Biochemical study

At 24 weeks, IVD samples from L6–L7, together with the adjacent AF and surrounding soft tissue, were resected and fixed in 20% formalin for the determination of PG content. In a preliminary experiment, PG content was slightly reduced with no statistical significance at 4 and 8 weeks; however, levels were significantly attenuated at 12 weeks^22^. After degreasing, 10 mg of NP were added to 0.05 mL of 3% NaOH in a 4-mL centrifuge tube, which was incubated at 40 °C in a water bath using a constant temperature vibrator for 3 h. Then, 2 mL of glacial acetic acid and 50 μL of 0.25% trypsin was added to the tube, which was further incubated at 50 °C in a water bath for 2 h. The enzyme mixture was transferred to a flask and divided into 10-mL aliquots. One milliliter of this solution was used for PG analysis according to the phloroglucinol method^20^. Absorbance of the reaction mixture was detected using a spectrophotometer (UV-2201; Shimadzu Corporation, Tokyo, Japan) at a wavelength of 558 nm, then transformed into PG content.

### Histopathological study

The IVD samples of L7–S1 collected from each subgroup were subjected to standard dehydration in baths of increasing percentages of alcohol, cleared in dimethylbenzene, embedded in paraffin, frozen for 20 min, and then serially sectioned into 5 µm-thick slices. The samples were stained with hematoxylin and eosin (HE), as well as safranin O by routine methods, and observed under a light microscope (Olympus Corporation, Tokyo, Japan). The cellularity and morphology of NP and AF were evaluated according to the Nishimura classification^43^ and all images were examined by another experienced histologist in a blinded manner. Safranin O binds to PG, so a stronger red staining with indicates a higher amount of PG, from which the concentration of PG can be determined to study the degeneration of NP cells.

### Immunohistochemical study

Each paraffin-embedded section was deparaffinized, dehydrated, and then boiled under high pressure and temperature in 0.01 mol/L sodium citrate for 3 min. Next, the sections were treated with 3% hydrogen peroxide for 10 min to block endogenous peroxidase activity. The sections were then incubated with the primary antibody (anti-collagen-1, 1:100, no. 2035; anti-collagen-2, 1:100, no. 2036; anti-TGF-β, 1:200, no. 2042; anti-MMP-1, 1:100, no. 2038; anti-MMP-3, 1:100, no. 2040, Bosheng Corp., Beijing, China) for 60 min at 37 °C. Then, the sections were incubated with an appropriate mouse-anti-goat secondary antibody (Dako Corp., Copenhagen, Denmark) at 37 °C for 20 min and colored with diaminobenzidine reagent (Dako Corp.). Finally, the immunohistochemical sections were counterstained with hematoxylin and observed under a light microscope. Images were saved using Image-Pro Plus software, version 7.0 (Media Cybernetics, Rockville, MD, USA). The mean grey values were used as indicators of the positive expression of collagen-1, collagen-2, and TGF-β, and the positive-expression rate were utilized as indicators of positively-stained MMPs. Visual fields of each image were randomly selected for further analysis.

### Statistical analysis

All statistical analyses were performed using IBM SPSS version 20.0 software (IBM Corp., Armonk, N.Y., USA). One-way analysis of variance was used to determine the significance of differences between all variables among the four groups. A least-significant difference post-hoc test was used to identify differences among groups. A probability (*p*) value of <0.05 was considered statistically significant.

## Supplementary information

Supplementary Information
