# Supplementary material for: The effects of simulated +Gz and microgravity on intervertebral disc degeneration in rabbits
Source: Sci Rep. 2019 Nov 12;9:16608. doi: 10.1038/s41598-019-53246-7 (PMC6851093; doi:10.1038/s41598-019-53246-7)
Supplement: Supplementary file 1 — Supplementary Information [file 41598_2019_53246_MOESM1_ESM.docx]

**Supplementary Information**

Title: The effects of simulated +Gz and microgravity on intervertebral disc degeneration in rabbits

The author list: Di Wu, Xi Zhou, Chao Zheng, Yu He, Lingjia Yu, Guixing Qiu, Zhihong Wu, Ji Wu, Yong Liu.

Figure 5. Immunohistochemical results observed by optical microscopy (×400) of the IVDs at L7-S1 of different groups at 24 weeks.


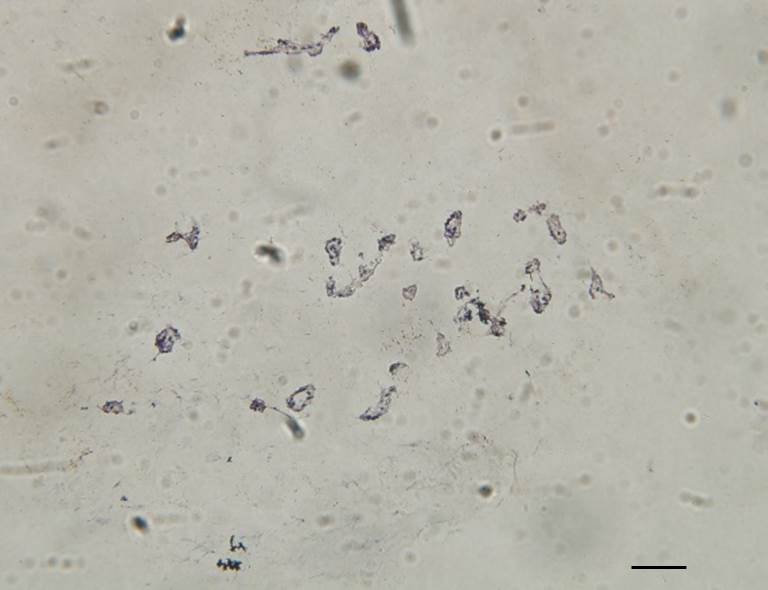


Collagen-1 in the control group (Scale bar = 10 μm).


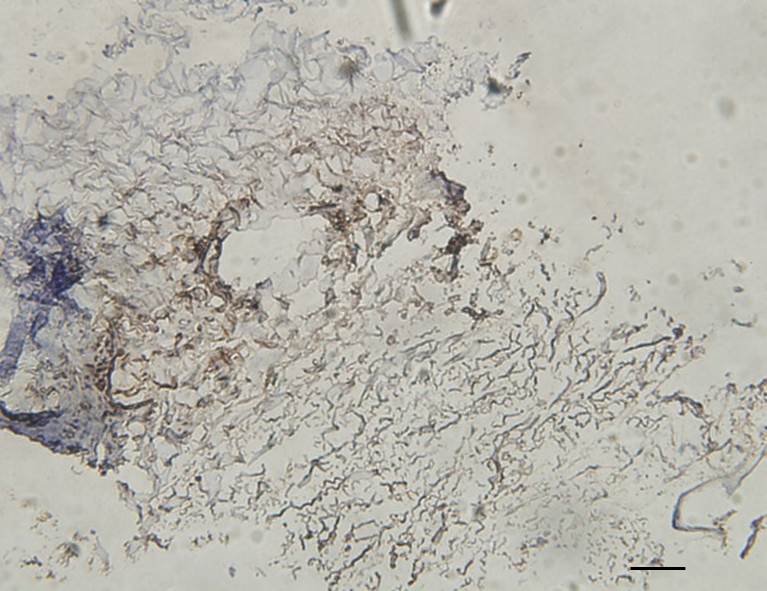


Collagen-1 in the microgravity group (Scale bar = 10 μm).


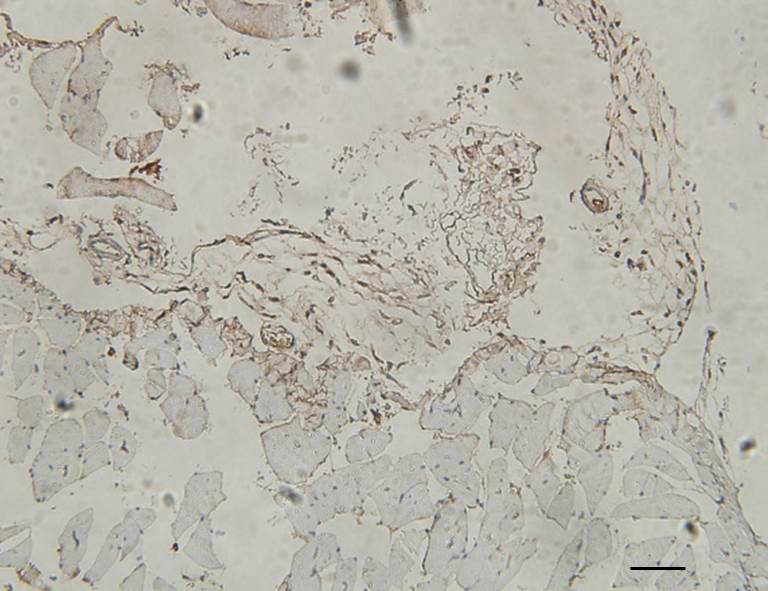
 Collagen-1 in the +Gz group (Scale bar = 10 μm).


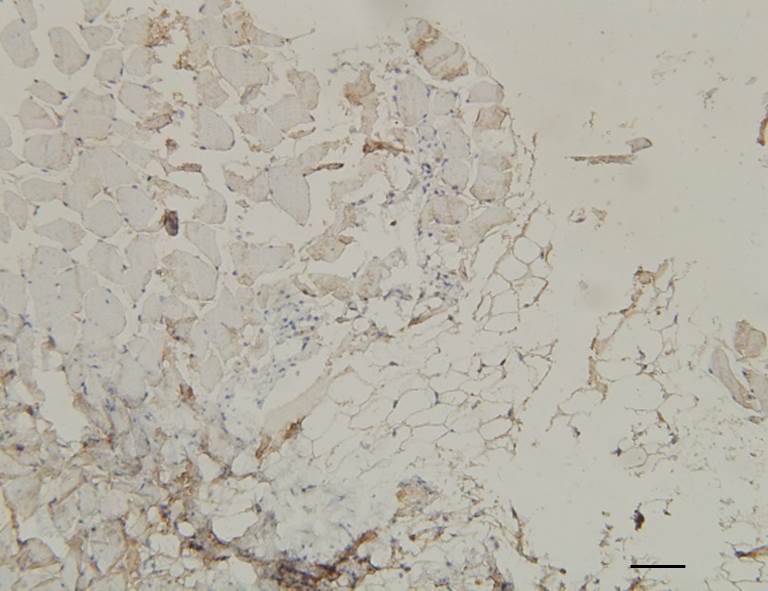
 Collagen-1 in the mixed group (Scale bar = 10 μm).


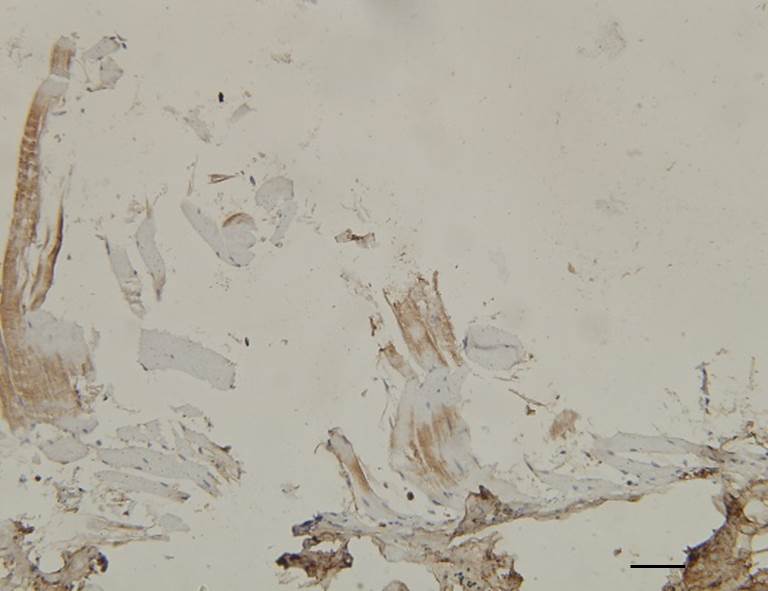
 Collagen-2 in the control group (Scale bar = 10 μm).


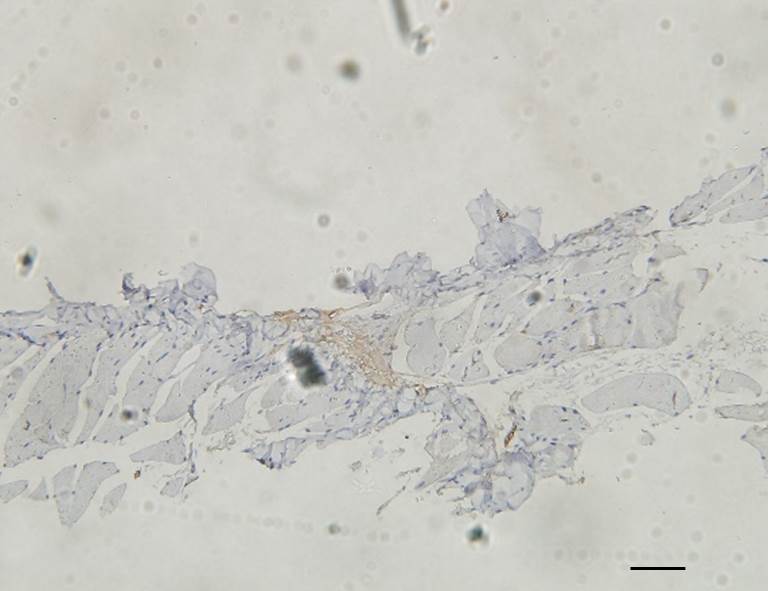


Collagen-2 in the microgravity group (Scale bar = 10 μm).


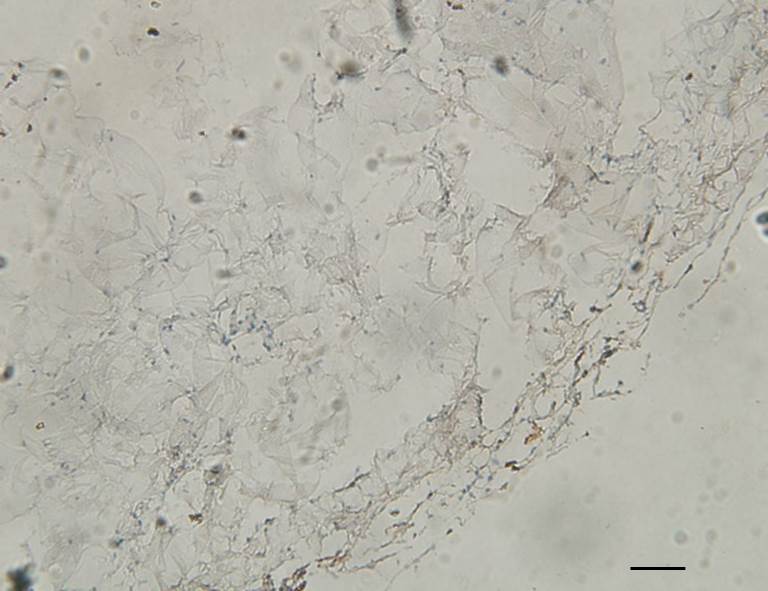
Collagen-2 in the +Gz group (Scale bar = 10 μm).


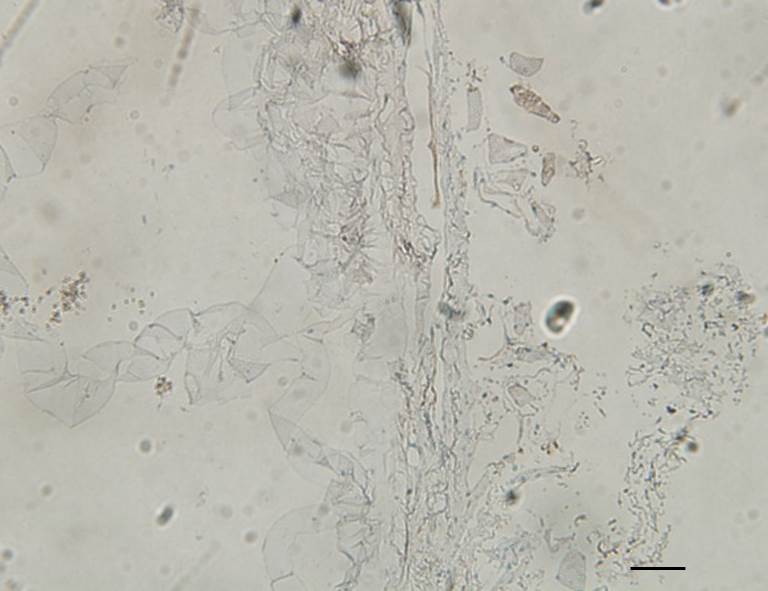
Collagen-2 in the mixed group (Scale bar = 10 μm).


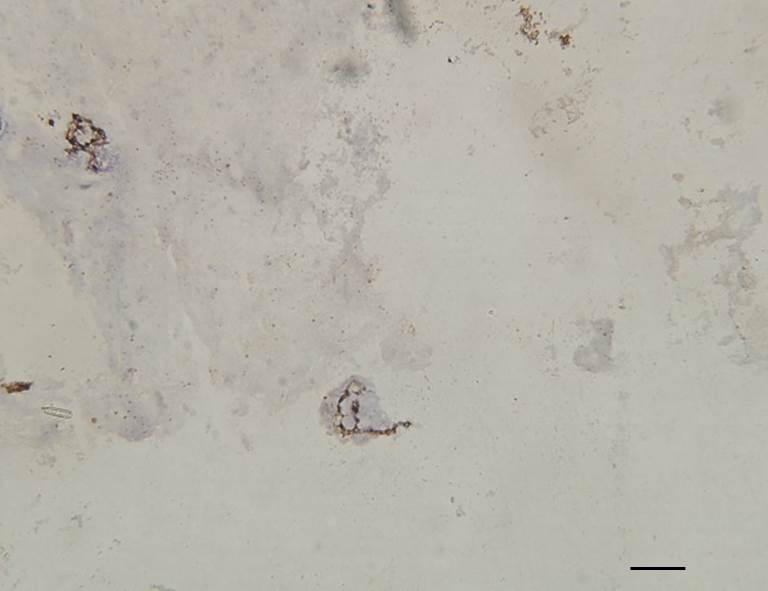


TGF-β in the control group (Scale bar = 10 μm).


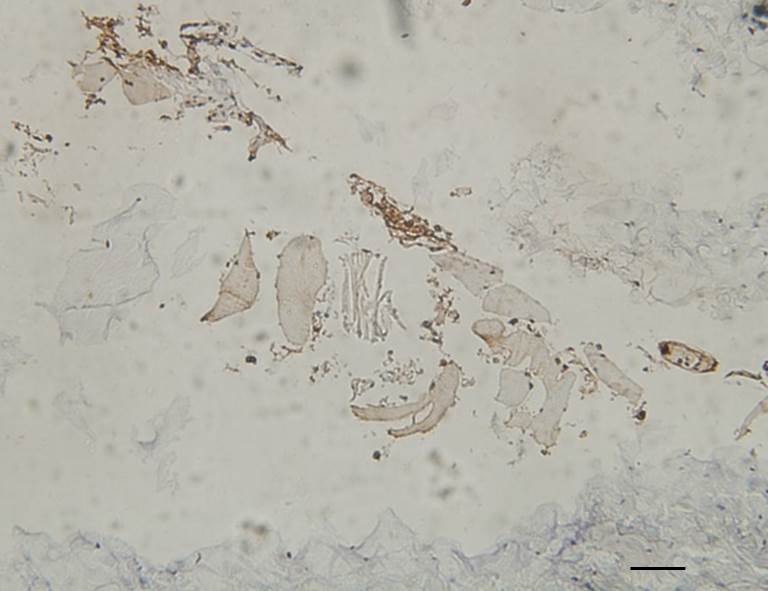


TGF-β in the microgravity group (Scale bar = 10 μm).


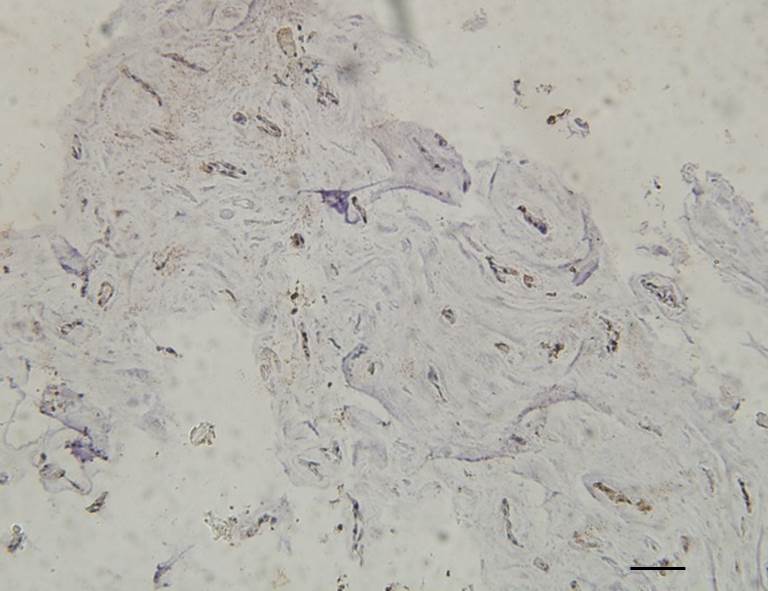


TGF-β in the +Gz group (Scale bar = 10 μm).


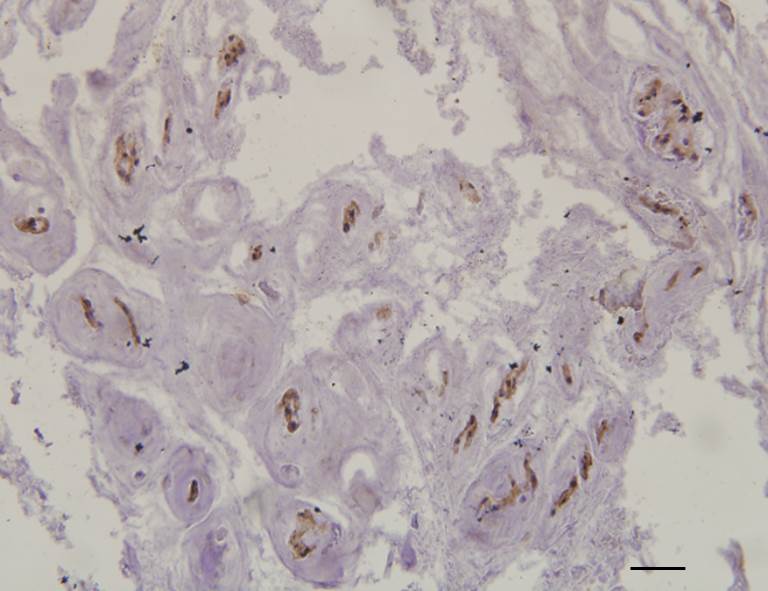


TGF-β in the mixed group (Scale bar = 10 μm).


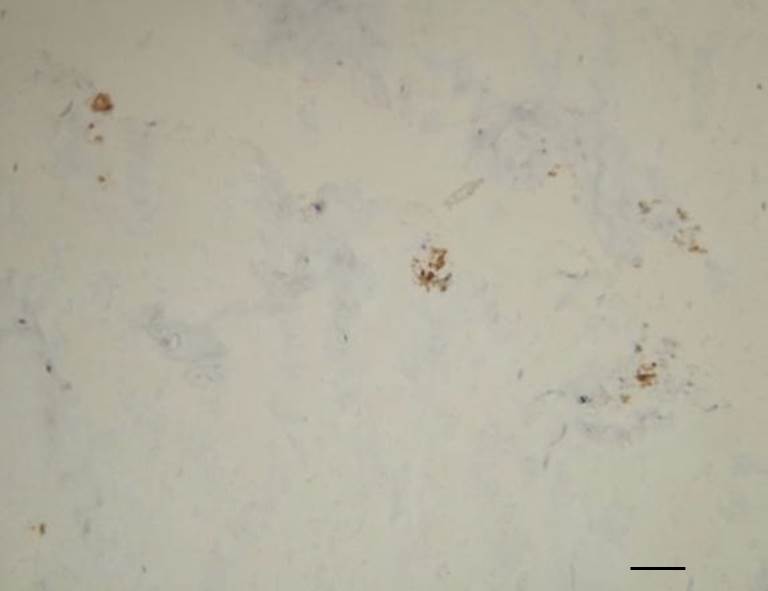


MMP-1 in the control group (Scale bar = 10 μm).


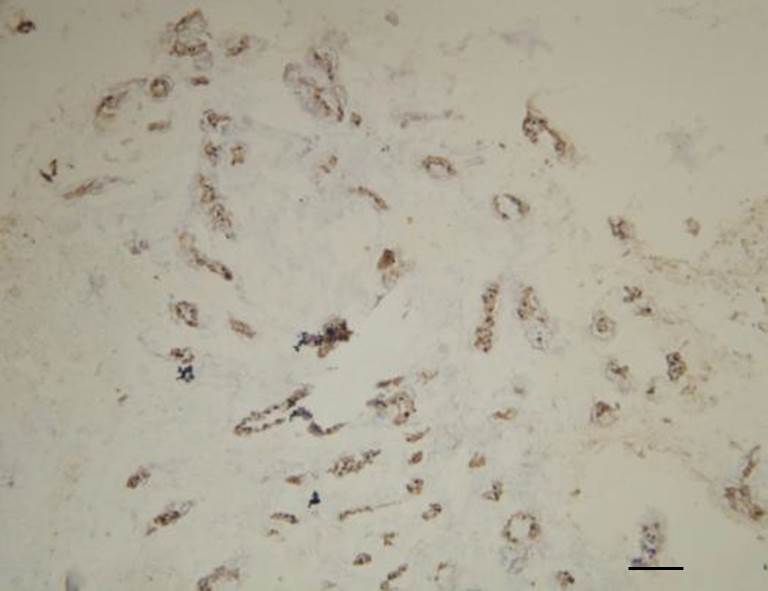


MMP-1 in the microgravity group (Scale bar = 10 μm).


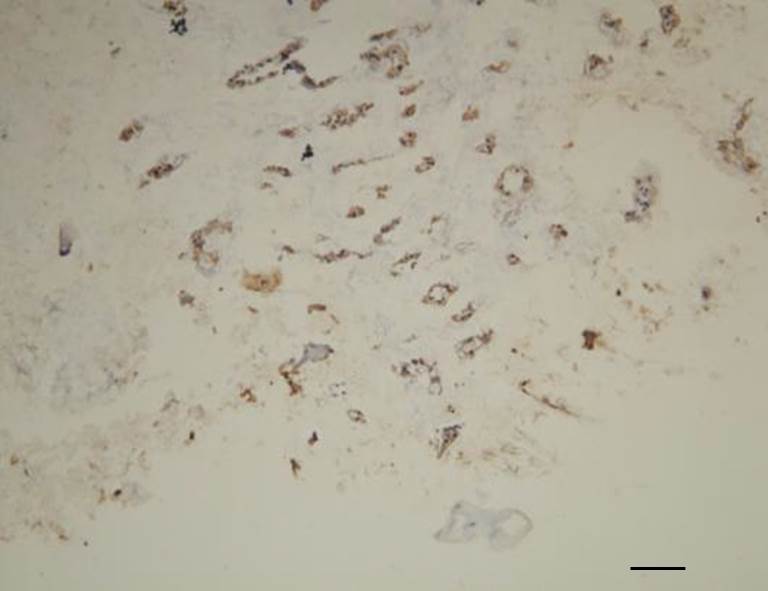


MMP-1 in the +Gz group (Scale bar = 10 μm).


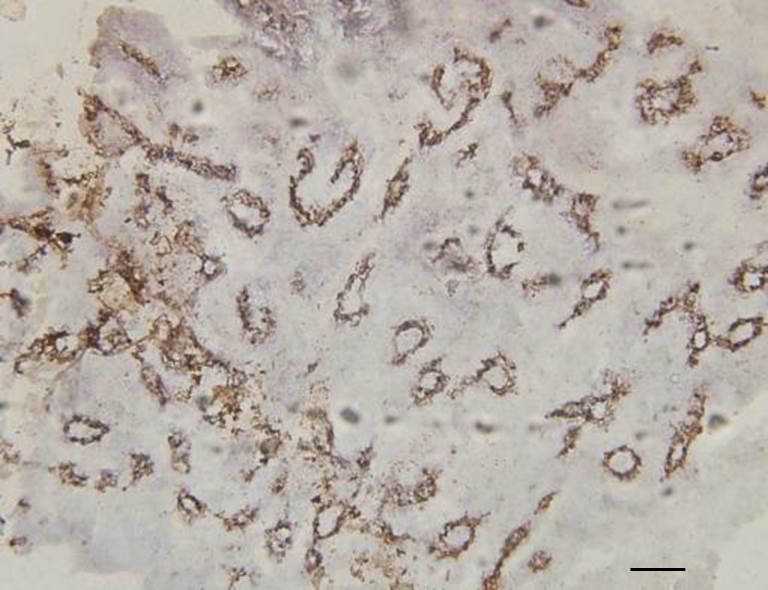


MMP-1 in the mixed group (Scale bar = 10 μm).


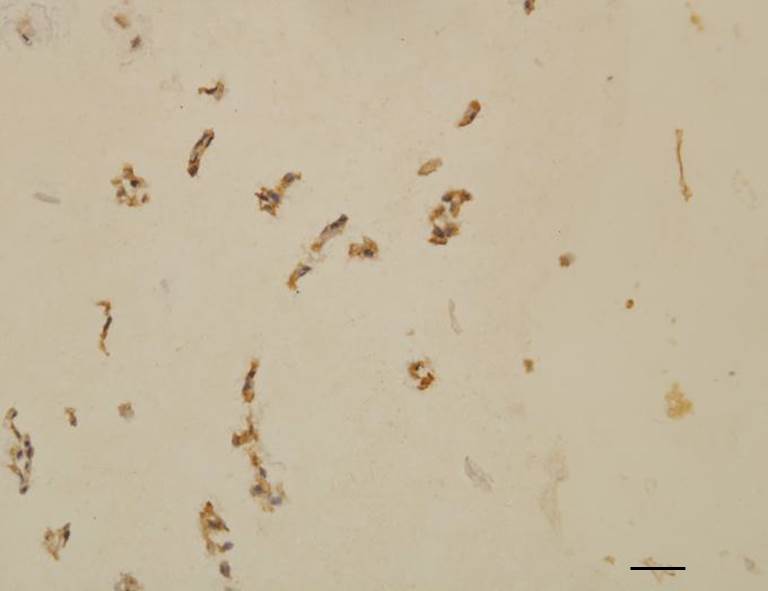


MMP-3 in the control group (Scale bar = 10 μm).


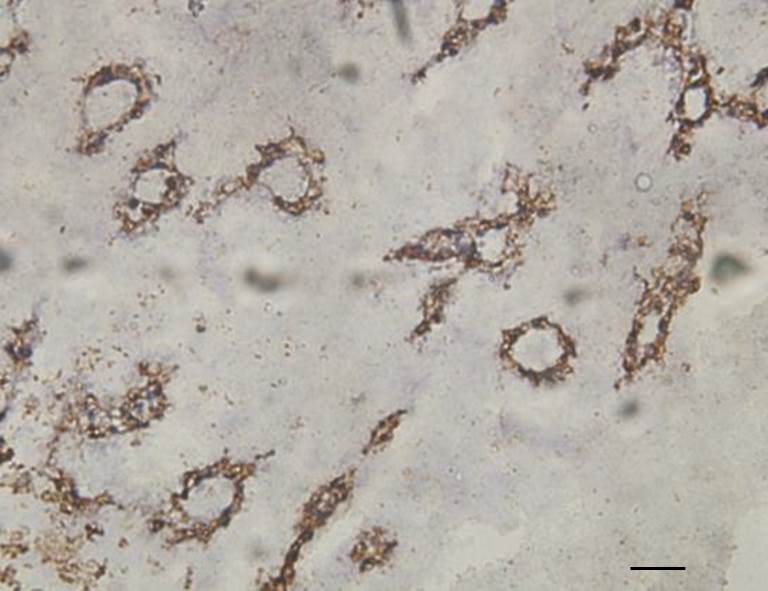


MMP-3 in the microgravity group (Scale bar = 10 μm).


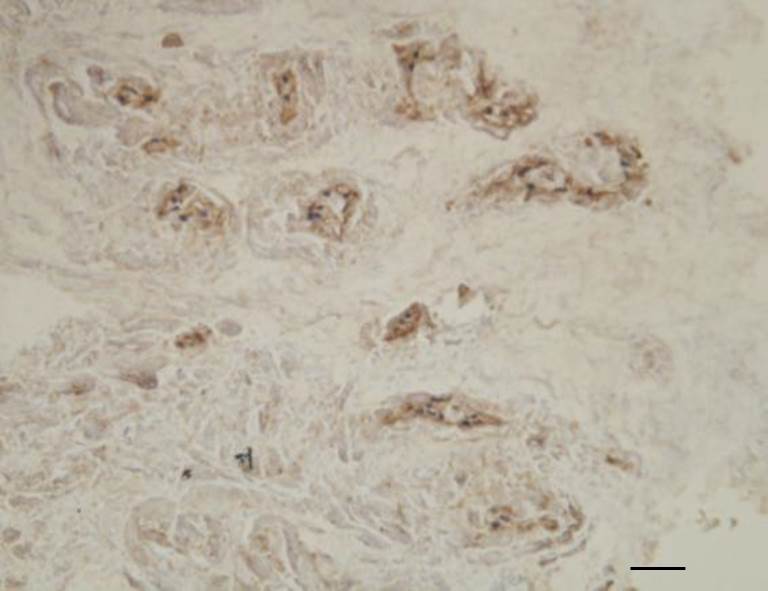


MMP-3 in the +Gz group (Scale bar = 10 μm).


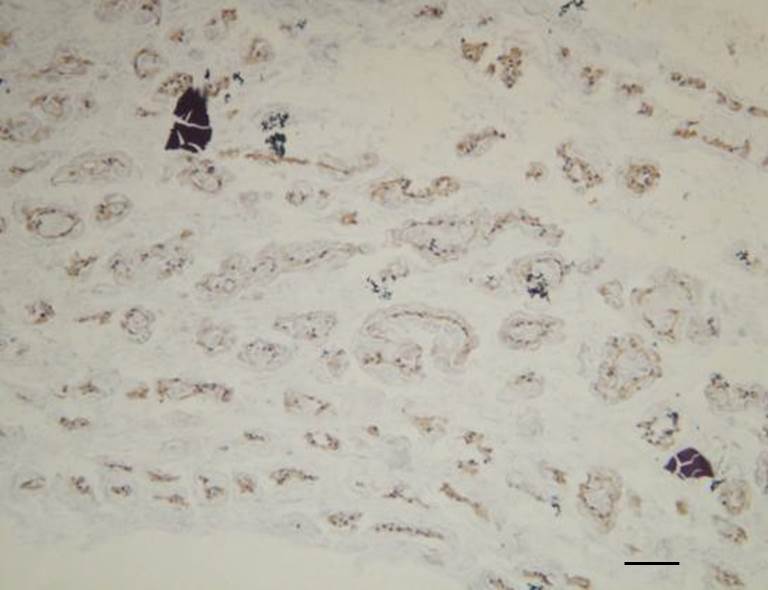


MMP-3 in the mixed group (Scale bar = 10 μm).
